# Appropriateness and Impact of a Vocal Cord Vibration Switch for Children with Complex Communication Needs: Case Series

**DOI:** 10.2196/75626

**Published:** 2026-03-31

**Authors:** Leslie Mumford, Denise Guerriere, Tom Chau

**Affiliations:** 1 Bloorview Research Institute, Holland Bloorview Kids Rehabilitation Hospital, 150 Kilgour Road, Bloorview Research Institute, Toronto, ON, M4G1R8, Canada, 1 4164256220 ext 3515; 2 Institute of Health Policy, Management and Evaluation, University of Toronto, Toronto, ON, Canada; 3 Institute of Biomedical Engineering, University of Toronto, Toronto, ON, Canada

**Keywords:** complex communication needs, augmentative and alternative communication, vocal cord vibration switch, children with disabilities, access technology

## Abstract

**Background:**

Communication is an essential component of participation. Communication impairment restricts full participation for children who have unintelligible speech. A vocal cord vibration switch offers an avenue for meaningful interaction to children who cannot rely on speech or voluntary limb movement but have some control of their vocal cords. Previous evaluations of the vocal cord vibration switch have been conducted primarily with adults and adolescents. However, implementation of a vocal cord vibration switch with younger, school-aged children in their natural environmental contexts can potentially foster the development of early communication skills.

**Objective:**

This case series evaluated the appropriateness and impact of a vocal cord vibration switch, the “Hummingbird” (Holland Bloorview) with school-aged children who have complex communication needs and their mothers and teachers, using an individualized, collaborative, and iterative assistive device implementation protocol.

**Methods:**

The Hummingbird was evaluated with 3 school-aged children, across educational and health-related contexts, over a 2-year period. Baseline, midterm, and final assessments took place at home or school in the first year with a follow-up assessment in the second year. In addition to field observations and device performance assessments by the research team, feedback from mothers and teachers was collected via questionnaires (Pictorial Children’s Effort Rating Table, Quebec User Evaluation of Satisfaction with Assistive Technology, and the Family Impact of Assistive Technology Scale) to ascertain the Hummingbird’s appropriateness and its impact.

**Results:**

Appropriateness data indicated the suitability of the Hummingbird across settings. Compared to the child’s prestudy devices, mother and teacher participants reported that the physical effort required by all 3 children to use the Hummingbird was lower (scores on the Pictorial Effort Rating Table decreased from 8 and 11 to scores of 4‐6). The switch efficacy assessment of the Hummingbird indicated moderate-to-high specificity and high sensitivity at midterm and high sensitivity (0.91‐0.94) and specificity (0.92‐1) at final assessments. Total satisfaction scores increased from baseline (prestudy device) to the 2-year assessment for all 3 children. While data on the impact on family and communication were incomplete for 1 participant, generally favorable effects were reported. The field notes underscored the value of an individualized protocol, where the implementation and evaluation phases were adapted to accommodate the health-related characteristics (eg, seizure disorder and sleep deprivation), evolving school contextual factors (new school and teacher), and unique family environments (involving the child participant’s toddler-sibling in Hummingbird sessions).

**Conclusions:**

Overall, the Hummingbird was appropriate across home and school settings for our case study participants, all of whom had complex communication needs. The device was well-received by children and their mothers or teachers, providing an effective, setting-agnostic option for communication support. Modifications to both the device and its implementation process were required to address unanticipated health, family, and school challenges.

## Introduction

Communication impairment is associated with limited participation in children with complex disabilities [1,2]. Participation, defined as the engagement in all facets of life, has been identified as an essential outcome for children with disabilities [3,4]. Full and effective participation demands being on an equal basis with others and transcends home, school, health, and community environments. This engagement facilitates quality of life [5] and development [6], allowing children to realize their potential [4]. While communication is one of the core components of participation, communicative impairment has been associated with participation restrictions for children with unintelligible speech [1,3,4].

Augmentative and alternative communication (AAC) systems can enhance communication and, ultimately, participation for children with complex needs [7,8]. Electronic AAC systems produce speech via technology external to the child’s body [9,10]. For children who have physical limitations that restrict direct access to electronic AACs via touch, an accompanying access technology is necessary. An access technology consists of a sensor that captures the child’s intent from a physical movement, vocalization, or physiological indicator and a processing unit that translates the detected intent into a corresponding control signal [11]. A vocal cord vibration switch, one type of access technology, provides an avenue for meaningful interaction to children who cannot rely on speech or voluntary limb movement but have some control of their vocal cords. This switch consists of a sensor, positioned over the vocal cords, to detect periodic vibrations resulting from the child’s vocalizations, which are translated into an output signal for computer control [12].

Previous evaluations of the vocal cord vibration switch have been conducted primarily with adolescents, adults, and children older than 8 years [12-15]. Intervening with AAC as early as possible allows for the opportunity to foster children’s communication skills and establish their participation potential earlier in their development [10]. School-aged children, particularly those with complex disabilities, rely heavily on their family members and teachers to facilitate social engagement. As parents and teachers play an integral part in these children’s participation by defining the opportunities to participate [3], engaging them in the implementation is essential [16]. Furthermore, in previous studies, the vocal cord vibration switch was evaluated over trajectories of 4 months or less. Offering children and their communication partners more time to use the vocal cord switch with support may encourage device adoption [17,18] and enable an evaluation of their experiences later in their trajectory. Finally, these assessments focused on the function of the Hummingbird and did not address the environments in which it would be used.

Accordingly, the purpose of this longitudinal case series was to evaluate the appropriateness and impact of the implementation of a vocal cord vibration switch with school-aged children who have complex communication needs in their natural environments (home and school), based on a collaborative, iterative, child- and family-centered protocol. Mothers’ and teachers’ perceptions of effort exertion, satisfaction, engagement, and psychosocial impact were measured. Device appropriateness was also assessed by the research team, and observations of process and progress were recorded in field notes. Temporal changes were measured across contexts (home and school) over a 2-year assessment period to allow for ongoing support and assessment after the initial introduction of the vocal cord vibration switch.

## Methods

### Overview

**Figure 1.**
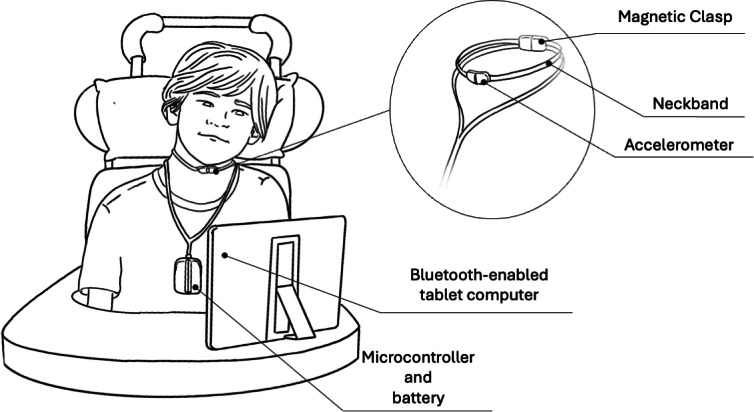
The Hummingbird device worn by a child, interacting with a computer through intentional vocal fold vibrations. The neckband is fastened by a magnetic clasp around the child’s neck. A control signal is transmitted to the tablet computer whenever an intentional vocal fold vibration is detected.

In this 2-year multiple case series, a vocal cord vibration switch, the “Hummingbird,” was introduced to 3 school-aged children, guided by the Assistive Technology Delivery Protocol (ATDP) [19,20]. The ATDP informs the introduction and adoption of assistive technology for children who have severe disabilities and encourages device adoption by focusing on the compatibility of the technology with the needs of the users, while ensuring standardized training protocols, multidisciplinary inclusion, and an iterative assessment process. The Hummingbird consists of a dual-axis accelerometer, which is held in place on the child’s neck proximal to the vocal cords using a neckband. The sensor is connected to a microcontroller box, where input vocal cord vibration signals are monitored and analyzed to detect short- or long-duration intended sounds (ie, voiced sounds or hums; Figure 1).

**Figure 2.**
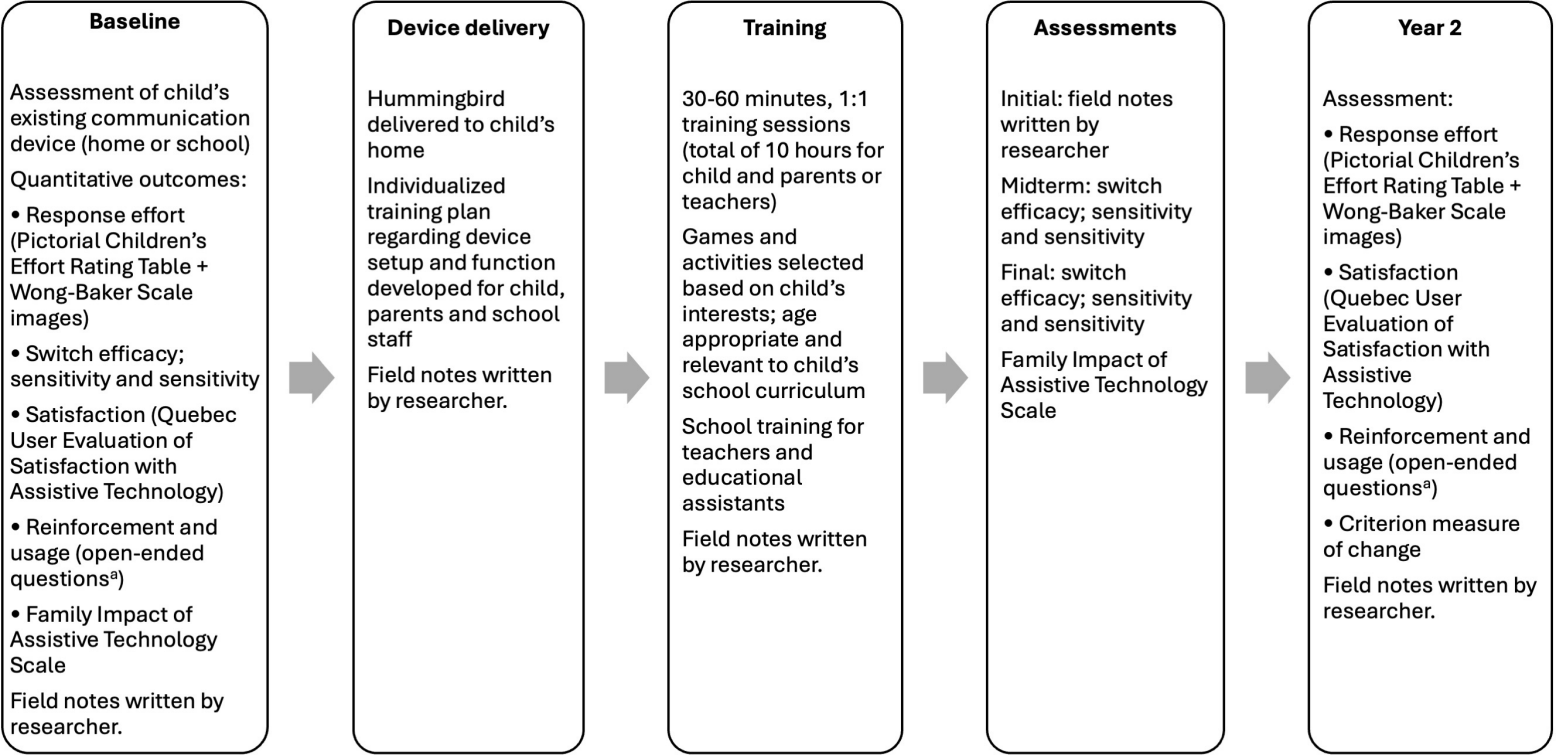
Overview of implementation and assessment sessions. ^a^Open-ended questions for reinforcement: On average, how many attempts does the child make to activate the Hummingbird before he or she is successful? Is there any delay between when the Hummingbird is activated and when the child perceives a response from the technology? Is there any delay or lack of response from the communication partner?

For this implementation, assessments across multiple time points were conducted, including a follow-up assessment (year 2; Figure 2). In the baseline session, assessments of the child’s existing communication device were conducted at home or the participant’s school, according to the preference of the family. Following the baseline assessment, the Hummingbird was delivered to families at home, and an individualized training plan was developed by the research team to include the children’s teachers and parents. The training plan was informed by the child’s family, caregivers (personal support workers, nurses, or additional family members), and hospital-based occupational therapists or speech-language pathologists. Device training for the children occurred in 30 to 60 minute, one-on-one sessions for a total of 10 hours over a 10 to 20-week time frame and involved games and activities that were age-appropriate and of interest to the child. A school integration phase consisted of training for teachers and/or educational assistants. No researcher involvement occurred in year 2 except for a check-in to determine if the children were still using the Hummingbird and to complete a subset of the outcome measures.

### Ethical Considerations

The study was approved by the research ethics board of the Holland Bloorview Kids Rehabilitation Hospital (eREB0291) and each of the participating school boards. To be eligible for the study, a participant had to meet the following criteria: (1) aged at least 4 years, (2) did not have an access pathway or had an existing access pathway that was deemed inappropriate as per International Organization for Standardization (ISO) 9241‐9, (3) understand cause and effect, and (4) have voluntary control over their vocal cords in a manner that generated a measurable vibration.

Additionally, one of the parents (or guardians) of the participant as well as his or her teacher had to possess English competency at no less than a grade 6 level and had to commit to the training workshops.

Participants were recruited by self-referral via the hospital website or through a therapist or teacher. A participant was deemed competent to provide consent if the participant had a reliable access pathway associated with a communication interface (AAC device, iPad with AAC application, or computer with AAC software) that allowed him or her to express his or her complete understanding or could use partner-assisted scanning to express his or her full understanding.

If the potential participant was deemed competent to provide consent, at the initial meeting, the principal investigator or co-investigator explained the study to the potential participant and his or her legal guardian. The potential participant was subsequently asked to explain to the researcher the purpose of the study and his or her involvement in his or her own words using his or her current mode of communication. If the potential participant appeared to understand and was able to provide responses with reasonable accuracy for all questions, he or she was asked to sign consent forms for participation in the study. If the potential participant could not provide answers that were accurate with his or her current mode of communication, he or she was asked to provide assent, and his or her legal guardian was asked to provide written consent. Assent was obtained by both a researcher and the family in order to ensure that the participant understood that he or she could opt out at any time. The participant was also reminded on a weekly basis that he or she did not have to participate if he or she did not want to.

If the potential participant did not meet competency criteria, the consent process followed as described above except that the participant was asked to provide assent, and his or her legal guardian was asked to provide written consent. Where applicable, the participant’s teacher and educational assistant or assistants provided written informed consent to participate in structured communication partner training. All participants were compensated for their time according to institutional research ethics board guidelines.

### Appropriateness and Impact

Four concepts were measured to evaluate the appropriateness of the vocal cord switch: (1) response effort; (2) switch efficacy; (3) mother or teacher satisfaction; and (4) reinforcement and usage. Response effort refers to the amount of physical and cognitive effort required to use the Hummingbird [19]. It was measured using a modified Pictorial Children’s Effort Rating Table (PCERT), consisting of effort descriptors, and a 6-level scale ranging from 0 (very easy) to 5 (very hard), with total scale scores ranging from 0 to 20 [21] and the Wong-Baker scale images [22]. The PCERT has yielded valid and reliable measures of effort with typically developing children [21,23]. In previous studies, scale scores have been moderately to highly correlated with objective measures of effort (Pearson *r*=0.54‐0.87), demonstrating concurrent validity of the scale at various levels of effort intensity [21], and the scale has demonstrated sufficient test-retest reliability (intraclass correlation coefficient=0.77) [23]. The PCERT was modified to use the images from the Wong-Baker pain scale, as cartoon faces ranging from happy to sad were considered more meaningful to children in wheelchairs than depictions of a child climbing stairs.

Switch efficacy represents a measure of effectiveness as defined by ISO 9241‐9; it was assessed using a switch-accessible game that required low cognitive effort and had unambiguous correct and incorrect responses for both the mother or teacher and the child [18]. The game also provided clear auditory and visual feedback to the child. Each child had 3 training sessions with the game to familiarize themselves with the task. Switch efficacy was based on researchers manually recorded data during the game and calculated using two equations: (1) specificity=true negatives/(true negatives+false positives); and (2) sensitivity=true positives/(true positives+false negatives). The specificity of a vocal cord vibration switch reflects the extent to which the device rejects the user’s unintended vocal sounds (eg, a cough) while the sensitivity is the device’s ability to correctly identify intended vocalizations. Based on previous evaluations of the efficacy of the Hummingbird with comparatively older children (aged >9 y), we anticipated that the 3 school-aged children in our series would achieve similar performances of specificity between 90% and 100% and sensitivity between 65% and 80% [13-15].

The Quebec User Evaluation of Satisfaction with Assistive Technology (QUEST 2.0) was used to assess caregivers’ satisfaction with assistive technology on a 5-point Likert scale ranging from 1 (not satisfied at all) to 5 (very satisfied). A total scale score and 2 subscale scores (Assistive Device and Services) are generated [24,25]. The QUEST 2.0 has been used extensively with clients of all ages, and it has been used to elicit parents’ [26] and health professionals’ [27] perceptions of their satisfaction with their child’s assistive technology. Psychometric evaluations of the QUEST 2.0 have yielded acceptable levels of internal consistency (Cronbach α=0.74‐0.79) [28]. Furthermore, moderate correlations between subscales of the QUEST 2.0 and the Psychosocial Impact of Assistive Devices Scale [29] have been reported (Pearson *r*=0.34‐0.45), supporting the concurrent validity of the scale [30].

To evaluate the performance of the Hummingbird, the rate of reinforcement (attempts required to activate the Hummingbird) and the immediacy of reinforcement (delay from technology or communication partner) were assessed by asking mothers and teachers open-ended questions (Figure 2). In addition, technology usage was determined by asking them to estimate the frequency of usage of the Hummingbird. Responses were recorded by the researcher.

The impact of the Hummingbird was assessed using two questionnaires. The Family Impact of Assistive Technology Scale (FIATS-AAC) detects the multidimensional effect of assistive device use on families with children with disabilities [31]. The purpose of this parent-reported questionnaire is to evaluate the functional effects of AAC systems on the lives of children and their families. It consists of 89 items, comprising 13 domains (7 child-related domains and 6 parent-related domains). Item scores are averaged to yield a domain score between 1 and 7. Higher scores suggest positive functional effects within a specific domain, and overall total scale scores range between 13 and 91. It has high internal consistency (Cronbach α=0.91) and test-retest reliability (intraclass correlation coefficient =0.95) [32]. Furthermore, its concurrent validity was supported as demonstrated by moderate agreement with a measure of family functioning (Pearson *r*=0.54) [33].

The Criterion Measure of Change Questionnaire [34] measures respondents’ rating of the change in their child’s communication since the commencement of Hummingbird with 3 ordinal categories (worse, no change, and better), as well as the extent and importance of this change on 7 levels ranging from a score of 1 (“a tiny bit”) to 7 (“a very great deal”).

### Field Notes

In addition to the administration of the quantitative measures, field notes were written. These notes, which were constructed by the researchers after each interaction with the children, mothers, and teachers, were unstructured and included observations of: the performance of the technology and the child, study and child or mother or teacher progress, communication with the child’s mothers, teaching staff and clinicians, and protocol implementation. The notes were compiled and analyzed using a manifest content analysis [35,36], which relies on the face value of the words written by the researcher and requires the use of preconceived categories for initiating and guiding the analysis. These categories included the following: the child’s health and communication characteristics and needs, the benefits and disadvantages of the child’s existing communication technology at study entry, the child’s experiences with using the Hummingbird (child’s likes and dislikes, successful strategies for encouraging use, and activities performed), challenges in using the Hummingbird specific to the child and mother or teacher, problem-solving, and device training and Hummingbird use at year 2. The field notes were grouped into units (sentences or phrases written by the researcher) within each of these categories, and then further organized by concepts (eg, successful strategies) within the category (eg, experiences with using the Hummingbird). This deductive approach allowed for a rich description and provided context to the child’s use of the Hummingbird at their home and school, over time.

The purpose of the field notes was to provide context, that is, to identify aspects of the technology or the environment that can improve and support the child’s use of the technology. They are useful beyond the study context in that they provide perspective on the aspects of the implementation and evaluation process that may need to be customized for individual children in future studies.

The ATDP [19,20] was used as a framework to design and guide the implementation and assessment process for this case series. The choice of questionnaires and the conceptual anchors for the field notes were guided by the ATDP, specifically the extent to which the Hummingbird meets the overall communication needs of the children. According to this protocol, successful Hummingbird adoption relies on the extent to which the technology is appropriate for each individual child. Appropriateness is defined as the efficiency, effectiveness, and satisfaction associated with the Hummingbird. Furthermore, the impact of the Hummingbird on the child and the family is essential to its success. The extent to which the Hummingbird is aligned with the characteristics of the children, mothers, and teachers is ultimately dependent on achieving a good match between the child and the Hummingbird within their given environment (school and home). The likelihood of this match is strengthened by the guidance of the ATDP.

## Results

Herein, the field notes are presented among the results from the quantitative measures to provide context and detail. The clinical and demographic characteristics of the 3 child participants appear in Table 1.

**Table 1.** Demographic characteristics of child participants.

Child	Age^a^ (y)	Sex	Diagnoses	School	Sensory function (vision; hearing)	Cognitive function
Eve	7	Female	Hydrocephalus	Special education classroom	Some impairment; no impairment	Some impairment
Jameel	5	Male	Cerebral palsy spastic quadriplegia	Home-schooled	No impairment; no impairment	No impairment
Zach	6	Male	Global developmental delay and epilepsy	School	Cortical visual impairment; no impairment	Significant impairment

a At study enrollment.

### Field Notes: Participant Description and Rationale for Hummingbird Introduction

At the start of the study, Eve was aged 7 years and attended a congregated public school for children with physical disabilities. In school, she used button-type switches (BIGmack button device) [31] with her hands, requiring a lot of assistance, demonstrating little accuracy, and experiencing a lot of fatigue. Her fatigue increased as the day progressed. Eve could control her head movements but had difficulty maintaining a steady head position, which resulted in her head dropping forward or to the side. She would vocalize when excited, happy, or upset. Accompanied by facial expressions, these vocalizations were used by parents and teachers to attempt to interpret what she was trying to communicate. She was able to verbalize the words “mama,” “papa,” and “hi,” and communicate with facial expressions and sounds that only familiar communication partners could understand.

Jameel enrolled in the study at the age of 5 years and was home-schooled. At that time, he had been attempting to use a Tobii eye-gaze system [32], but the high tone of his muscles made it difficult for him to hold his head in one position, even with physical head support. As such, he found it challenging to maintain a dwell long enough for the eye-gaze system to activate the desired choice on the screen. Jameel had good voluntary control of his voice. The Hummingbird was considered a potentially appropriate device for him, as it does not require head stability, and he possessed the vocal control for activation. It was hoped by his parents that he would be able to combine the eye-gaze system and the Hummingbird.

Zach was aged 6 years and attended a specialized school for children with disabilities at the start of the study. It was difficult for Zach to control his limbs and head, and he experienced frequent seizures. He had previously tried to use button-type switches (BIGmack button device) by controlling them with his hand and head, but he found them too tiring. He was able to indicate ‘yes’ using a simple non-word vocalization (‘ah’). This vocalization had been used to elicit answers using partner-assisted scanning and a communication book. However, his family reported that the pictures in the communication book were challenging for him to discern due to his cortical visual impairment. Zach’s family expressed a desire for an appropriate technology for him so that he could demonstrate his comprehension and answer questions in the classroom, as it was otherwise difficult for him to display his capacity for learning and understanding class material. Zach did not have the first-year final assessment for the FIATS because he was not using the Hummingbird at home, only at school during the first year. The Criterion of Change was also missing for this participant.

### Field Notes: Observations During Sessions

Eve really enjoyed music and as such, it was used as the initial basis for her switch training. She began with errorless activities where she had to activate the Hummingbird to hear the next verse of a song and moved on to timed options where she would activate her Hummingbird to sing a repeating line from a song at the correct time. She often needed prompting to “wait” for the correct time to activate, but this need for prompting reduced over time. There were several interruptions to Eve’s participation in the study as she changed schools and school boards multiple times throughout the study. After consultation with one teacher, choice-making during circle time activities was chosen as the focus for classroom training for Eve.

Jameel progressed very rapidly from simple cause and effect exercises to games that required precise timing. At the start of the study, he was working with his mother (who was also his home-school teacher) and an educational assistant, with a home visit approximately once per week from a member of the research team. One of the main challenges for Jameel was finding activities that motivated him; he could easily become bored with an activity that was presented in a repetitive fashion. This made evaluation of the switch efficacy quite challenging—after just 1 or 2 trials of a game, he sometimes refused to play more. Despite his excellent performance with the Hummingbird, the study progressed more slowly for Jameel due to personal and family illnesses, leading to a hiatus of approximately 4 months. Even after the break, however, Jameel quickly regained proficiency in the use of the Hummingbird. In addition, Jameel’s family had been limiting their use of the Hummingbird to prevent Jameel’s toddler sibling from pulling and/or breaking the device wires. Jameel started using a wireless version of the Hummingbird, which became available midway through the study, and his use of the Hummingbird increased after this change. With the new wireless Hummingbird, Jameel was playing games in addition to controlling switch-activated toys that also engaged his sibling.

During Hummingbird training, Zach was motivated by music, astronauts and space, and any activities involving his family members. He was also motivated by helping others, so the research team would reframe simple games as him “helping” the character move through the story or activity. Auditory cues were used as often as possible to reduce his need to rely on his vision; bright colors on a dark background were used as these were easiest for him to see. Zach had sleep difficulties, which often required him to sleep during the day. Occasionally, he would fall asleep during his sessions or struggle to stay awake. On the days when he was well-rested, the time required for him to coordinate a response to scanning choices was 2 to 3 seconds, compared to 15 seconds when fatigued. In addition, he had frequent absent seizures. At times, the research team was unsure if he was processing his answer or temporarily unaware of the task due to seizure activity. Zach would begin a session in an alert state but could then become fatigued partway through. On good days, a quick “warm-up” using a short errorless game, followed by the actual task, seemed to give shorter response times. When he was fatigued at the start, the researchers tried to preserve his energy as much as possible for the actual task.

### Appropriateness and Impact: Response Effort (PCERT)

Table 2 presents effort levels, as reported by a mother or teacher, for the baseline assessment when the child was using their baseline or non-Hummingbird device and those for the year 2 assessment. The reported perceived effort required to use the Hummingbird by all three of the children was lower than the effort required for their previous device. The largest temporal change was for the physical effort required by Jameel’s mother, decreasing from the maximum effort of 5 to 2.

**Table 2.** Response effort scores^a^.

	Eve		Jameel		Zach	
	Baseline^b^	Year 2	Baseline^c^	Year 2	Baseline^b^	Year 2
Respondent	Teacher	Teacher	Mother	Mother	Mother	Teacher
Item						
Physical effort for user (child)	3	1	3	1	4	2
Cognitive effort for user (child)	2	1	0	1	4	2
Physical effort for caregiver (mother or teacher)	2	2	5	2	0	1
Cognitive effort for caregiver (mother or teacher)	1	0	3	1	0	1
Total (0‐20)	8	4	11	5	8	6

a By assessment time and item: 0 (very easy) to 5 (very hard), with total scale scores ranging from 0 to 20.

b BIGmack button device [37].

c Tobii eye-gaze [38].

### Hummingbird-Switch Efficacy

**Figure 3.**
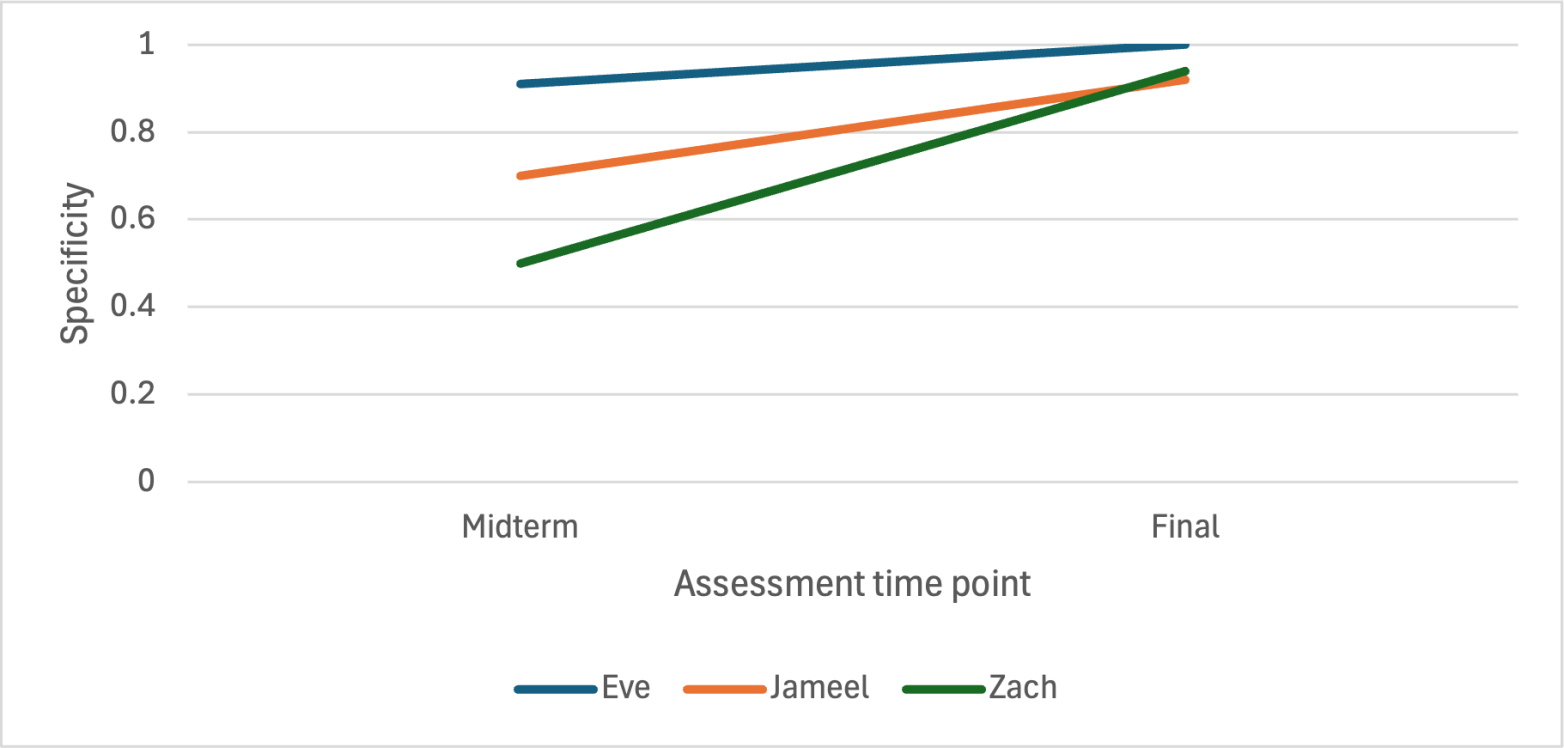
Switch specificity at midterm and final assessments.

Figures3  4 display switch efficacy at the midterm and final assessments for all 3 children. The Hummingbird demonstrated very high specificity (0.9 and 1) and sensitivity (0.8 and 0.9) for Eve at both the midterm and final assessments. For Jameel, specificity at the midterm was moderate (0.7) but increased in the final assessment (0.9). Finally, very high sensitivity at both midterm (0.9) and final (0.9) assessments was obtained for Zach, and a relatively low specificity at the midterm (0.5), which improved to a very high level for the final assessment (0.9).

**Figure 4.**
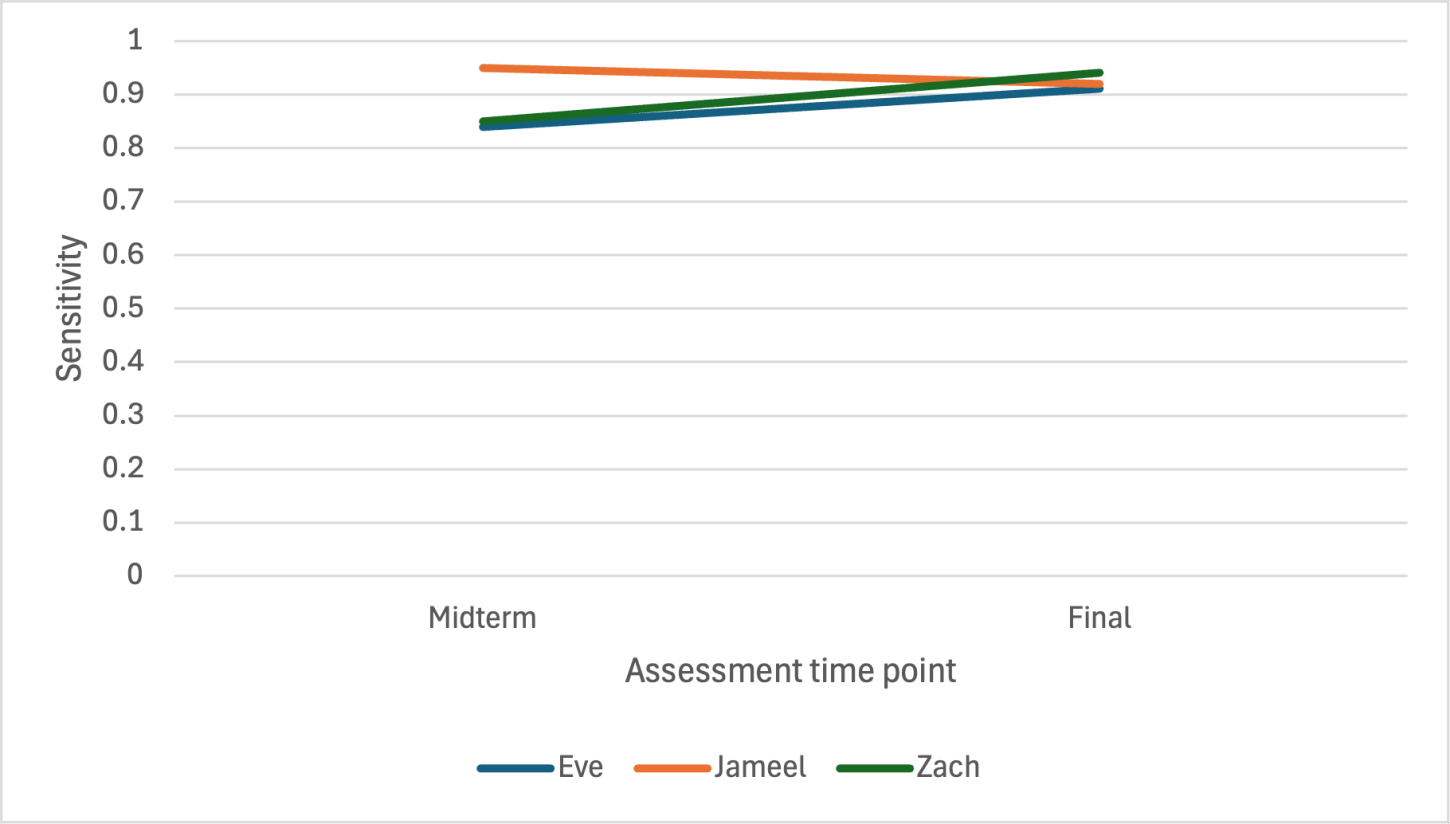
Switch sensitivity at midterm and final assessments.

### Quebec User Evaluation of Satisfaction With Assistive Technology

**Figure 5.**
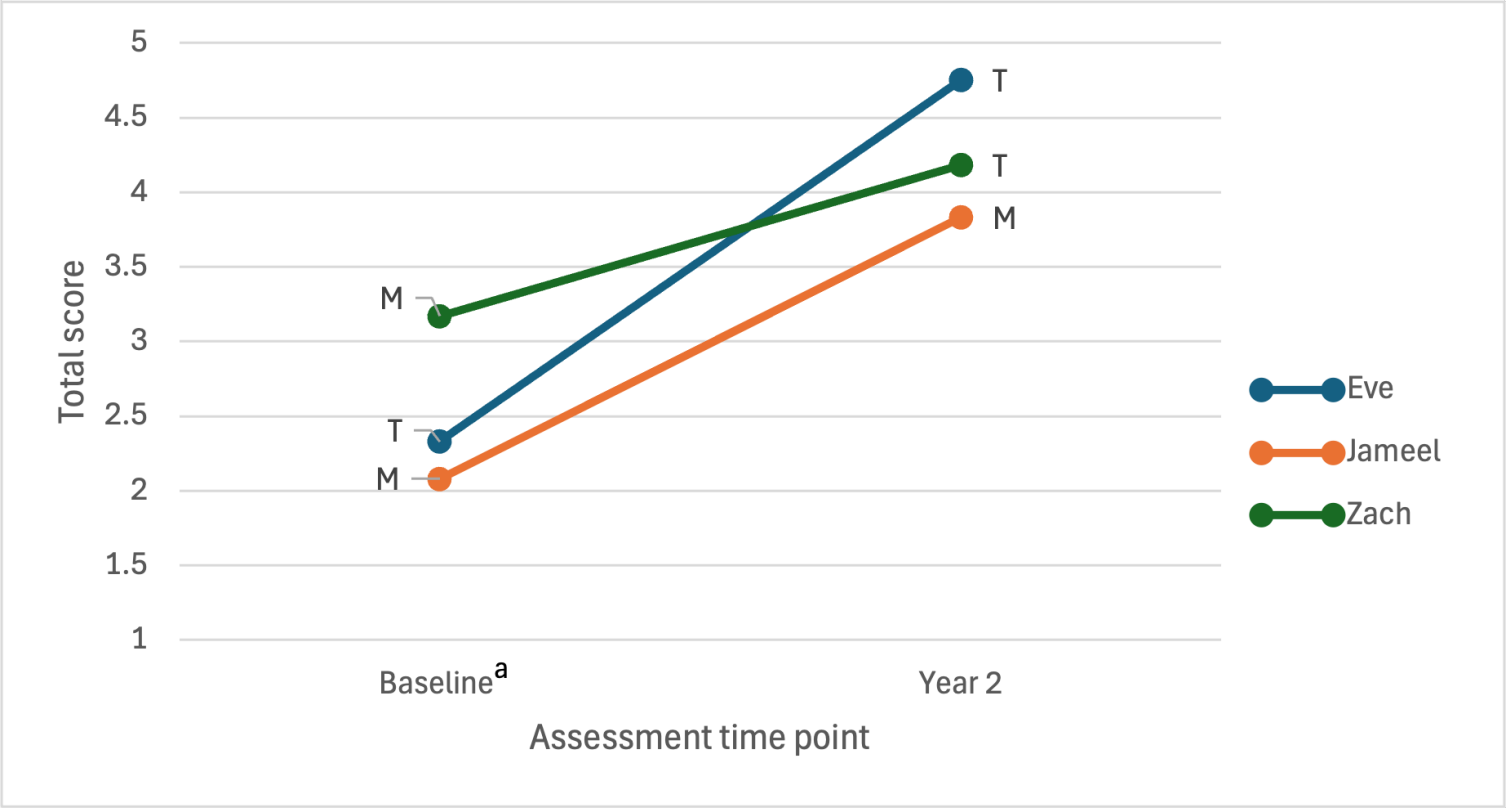
Quebec User Evaluation of Satisfaction with Assistive Technology (QUEST 2.0) total scores for baseline and year 2 (Hummingbird; 1=“not satisfied” to 5=“very satisfied”). ^a^Eve and Zach using the BIGmack button device, and Jameel using the Tobii eye-gaze at baseline. T: teacher report; M: mother report.

Satisfaction ratings by teacher or mother participants increased from baseline (using a non-Hummingbird device) to year 2 for all 3 participants (Figure 5). All 4 respondents (2 teachers and 2 mothers) rated “easy to use” and “effectiveness” as being one of the top 3 most important items. “Professional service,” “comfort,” and “dimensions” were each reported by 2 respondents.

### Rate and Immediacy of Reinforcement

The immediacy of reinforcement with the Hummingbird was consistent across the 3 participants, with no delay from the technology during their year 2 evaluation (Table 3). The usage of the Hummingbird, as reported by mothers and teachers, was lower than that of her previous device for Eve. Due to inconsistency in reporting, comparative usage is difficult to determine for Jameel and Zach. In Table 3, immediacy of response *from the technology* refers to whether there was a delay between activating the device and the intended output (ie, the Hummingbird responding via message output). On the other hand, immediacy of response *from the communication partner* refers to any delay between the time the child used the Hummingbird to communicate (eg, made a choice or requested something) and the time at which their communication partner acknowledged the message. In this latter context, no delay meant that the communication partner (eg, mother or teacher) acknowledged the message and desired action as soon as the child “says” it.

**Table 3.** Reinforcement and device usage at baseline and year 2 assessment.

	Eve		Jameel		Zach	
	Baseline (BIGmack)	Year 2 (Hummingbird)	Baseline (Tobii)	Year 2 (Hummingbird)	Baseline (BIGmack)	Year 2 (Hummingbird)
Respondent	Teacher	Teacher	Mother	Mother	Mother	Teacher
Usage	2 times per day for 30 minutes each Weekly total: 420 minutes	3 days per week for 25‐40 minutes each Weekly range: 75‐120 minutes	5 days per week for 10‐30 minutes Weekly range: 10‐150 minutes	Varied from: once daily for 5 days per week for months to not being used for months Weekly rate: 5	2 times per day at home and school Weekly rate: 14	Minimum 1 time per day for a few minutes to share a short message Weekly minimum: 35 minutes
Rate of reinforcement	On some days, 2 attempts and other days, 3‐4 attempts to hit switch—depends on mood+health	Activates every time	1 attempt for playing games Many attempts for communication	With practice, 2 attempts	Needs prompting or hand over hand to start, then uses without difficulty	1 attempt when device is working properly (ie, tuned to the user’s vocal fold vibration), otherwise several attempts
Immediacy of reinforcement	No delay	No delay from either technology or communication partner	Delay from technology when used for communication No delay from communication partner	No delay from technology	Delay in activating—quicker for the child to vocalize than hit the button	No delay

### Impact

Table 4 presents scores for Eve and Jameel for baseline and final assessments, as reported by their mothers. An increase in scores over time indicates gains (positive functional effects) in dimensions from baseline to final for Jameel. The “face-to-face communication” domain increased from 2.4/7 at baseline to 5.1/7 for the final assessment. The “doing activities” domain also increased from 2.6 to 3.8. No temporal change in the overall score was observed for Eve. Only 2 domains increased for Eve over time, namely, scores from 3.6 to 4.3 for “contentment” and scores from 3.8 to 4.3 for “family roles,” otherwise, her domain scores decreased (doing activities and security) or remained the same.

In year 2, the Criterion Measure of Change Questionnaire, which rates a child’s communication on 3 ordinal categories (worse, no change, and better), indicated that both Eve’s and Jameel’s communication was rated “better” than when they entered the study (baseline). The extent of this improvement was quantified as 2/7 and 4/7, and the importance of this change as 3/7 and 7/7 for Eve and Jameel, respectively.

**Table 4.** Family Impact of Assistive Technology Scale mean domain scores for Jameel and Eve over time, as reported by their mothers.

Domain	Jameel^a^		Eve^b^	
	Baseline	Final	Baseline	Final
Child-related				
Behavior	4.5	4.7	6	6
Caregiver relief	2.2	2.8	2.8	2.9
Contentment	3.4	4.9	3.6	4.3
Doing activities	2.6	3.8	4.2	3.8
Education	4.7	6	6	5.7
Energy	1.7	3	3.6	3.6
Face-to-face communication	2.4	5.1	2.8	2.6
Family-related				
Family roles	2.6	3.3	3.8	4.3
Finances	3.6	3	3.4	3
Security	3	3.9	2.3	3.1
Self-reliance	2.9	4.9	3.6	3
Social versatility	3.4	4.6	5	4.7
Supervision	2.1	3	2	2.6
Total score (range 13‐91)	39.1	53	49.1	49.6

a Home-schooled.

b Special education classroom.

### Field Notes: Observations at Year 1 Completion

By the end of the training phase, both Eve and her teacher were comfortable with choice-making activities. Eve really enjoyed vocalizing to talk or sing along, so the Hummingbird switch was only vigilant (ie, turned on) when she was being asked to make a choice. At those times, she could calm herself and wait for the option she wanted. This selective use of the Hummingbird was adopted to avoid the frustration of excessive accidental activations when she wanted to expressively communicate through her vocalizations. Eve would become excited for sessions and wanted to vocalize a lot. If she was in an excited state, the researcher would give her time at the beginning of the session to just play, activating her switch as much as she wanted, before asking her to relax and calm herself to do the activity. The teacher also waited patiently for her to reach her calm state before presenting her with choices.

By the end of the first year, Jameel was using the Hummingbird with the eye-gaze system and appeared to find it easier than using the Hummingbird alone with a scanning-based communication grid on a tablet. His mother reported that it was easier for him to combine the 2 access methods rather than relying solely on switch scanning with his communication grid on his tablet. He was exploring spelling and sentence building using predictive text, with the assistance of his educational assistant. Depending on his level of motivation for an activity, he could often use his set-up to select from multiple choices independently. At this point, Jameel’s toddler sibling had also spontaneously begun to model for him, attempting to get his attention by humming and pointing at the Hummingbird while saying, “My turn!”

Zach’s sleep difficulties and seizures made some of the evaluations difficult. When the research team was unsure whether Zach was processing his answer or temporarily unaware of the task due to seizure activity, more frequent indirect prompting was used when the time to produce a response seemed especially prolonged. Indirect prompting included presenting Zach with a visual cue or picture, telling him that something was expected (eg, “Now what?” or “What’s next?”) and using body language (eg, questioning hand motion). The team learned to carefully observe signs of increased effort, as Zach could start a session alert but become fatigued partway through. During some of the switch efficacy testing sessions, he struggled with fatigue and falling asleep; his behavior during those sessions did not reflect the team’s observations in other sessions.

## Discussion

### Principal Findings

This case series evaluated the appropriateness and impact of a vocal cord vibration switch with children aged 5 to 7 years across home and school settings, according to the ATDP. Despite some challenges related to the children’s health and environment such as fatigue, school changes, and illness, the results from this evaluation lend support for the use of the Hummingbird for school-aged children with complex communication needs. Overall, the implementation of the Hummingbird with 3 school-aged children was successful, and mothers and teachers responded positively to supporting its use at home and school.

### Appropriateness and Impact

In our case series, we observed higher sensitivity rates at both midterm and year 2, and similar percentages of specificity compared to 2 previous evaluations of the Hummingbird that were conducted over a shorter evaluation period with children who were primarily older. In 1 study with 2 children aged 5 and 9 years, the average specificity of the Hummingbird was 98.1% and 98.8%, respectively, and the average sensitivity was 73.3% and 80%, respectively [15]. Furthermore, previously reported sensitivity percentages for a child aged 12 years using the Hummingbird were 60% and 65% at 8 and 16 weeks, respectively, and specificity percentages similar to ours were reported at >90% [20]. Switch efficacy levels with children aged 5 to 7 years in our study are similar to those observed with older users, thus supporting the use of the Hummingbird with a variety of age groups. For the youngest participant, Jameel, who was aged 5 years at study entry, the Hummingbird correctly identified his intended vocalizations at a higher or similar rate as older users. As with any efficacy evaluation, the results can be affected by dynamic factors specific to the child or user at the time of any given assessment; for example, fatigue was such a factor for 1 participant in our case series as well as for 1 child in a previous implementation [15].

We observed that mothers’ and teachers’ response effort scores on the PCERT decreased for all 3 children from baseline, indicating that the perceived physical effort exerted by the child was lower when using the Hummingbird compared to their prestudy communication device. It is noteworthy that this decrease in effort was observed during the year 2 assessment when regular support and contact with the research team had concluded. Ease of use of an AAC has been reported in previous studies as an important determinant of parent satisfaction [39] and device use [40]. Furthermore, literature indicates that parents report device abandonment when the effort to use the device is too demanding [17].

Measuring parents’ and teachers’ satisfaction is essential, given that dissatisfaction with an AAC system is 1 factor leading to device abandonment [17]. Our satisfaction results suggest that the mothers and teachers in our evaluation had a higher level of satisfaction with the Hummingbird compared to the child’s previous device. Teachers’ and mothers’ satisfaction ratings increased from baseline to the year 2 assessment for all 3 children (scores ranging from 3.8-4.8). Similarly, in a previous case study, an individual aged 19 years also reported higher satisfaction with the Hummingbird (score=4.6) compared to a sound-based device [12]. Furthermore, in another assessment of the Hummingbird with a girl aged 12 years with cerebral palsy, similar QUEST 2.0 scores (4.0 and 3.5 at 8- and 16-wk postdevice introduction, respectively) were reported by her occupational therapist [20]. Our data thus support the applicability and usability of the Hummingbird with younger children than previously documented.

Device reliability is an important factor in preventing device abandonment [41]. In this case series, the quality of reinforcement evaluation indicated that, overall, the children were able to activate the Hummingbird more reliably, with fewer attempts and no delay compared to their previous prestudy device. The assessments during year 2 of the study revealed that all 3 children had continued to use the Hummingbird after the main study period.

Family impact scores on some of the FIATS domains for Eve and Jameel did not change from baseline, using their non-Hummingbird device, to the final assessment after months of using the Hummingbird. Overall, Jameel’s total scale scores indicated a moderate increase in the positive functional effects with the Hummingbird (from 40/91 to 53/91) and no change in total functional effects for Eve. Perhaps because Jameel was home-schooled, his mother may have observed more of an effect on family functioning. In addition, Jameel’s sibling started to play games with him while he was using the Hummingbird, which may have resulted in a greater overall impact on family functioning.

It is critical that AAC systems are designed for the individual to optimally facilitate communication and promote sustained user engagement [18]. In this study, personalization involved changes to the Hummingbird itself as well as to the process aspects of the protocol. The field notes provided insight into the characteristics of the children and their environments and permitted an iterative approach whereby adjustments to the training, implementation, and evaluation processes were made during the study. For example, the researcher (or researchers) explored different activities with each child; for example, Zach excelled when engaging in activities that involved helping people. A wireless version replaced Jameel’s original Hummingbird to address the needs of his family environment. These customizations encouraged engagement in subsequent sessions with the three children as well as offering a guide to the implementation process in future work.

Previous research in the broader AAC literature has supported the importance of training communication partners; effective partner training promotes the communication abilities of individuals with complex communication needs [18,42]. Despite this important element, most studies have excluded AAC training for parents and siblings of children with multiple disabilities.

### Value of Multisite Inclusion and Individualized Implementation Approach

Challenges are inherent in the implementation process of a device for children who have complex, co-existing conditions that compound their existing activity limitations. In our case series, the child participants differed from one another as they had a variety of health, social, family, and school context characteristics. The successful longitudinal implementation of the Hummingbird with these 3 school-aged children resulted from a comprehensive, individualized approach, including adjusting and modifying both the technology and the protocol, to meet the needs of the child and the child’s environment. For example, changing to a wireless device for Jameel was essential to encourage continued use of the technology when his brother was present.

As purported by the ATDP, training and support by the team ensure a child-specific approach that recognizes and adapts to the technology user, including elements of their physical and social or family environment. Of priority to our implementation was the inclusion of key players from each of the child’s environments. Furthermore, based on the ATDP, device training and implementation were individualized and embedded into the participants’ daily lives and natural environments, while evaluation was semipersonalized, such as affording the child the choice of a game. Inclusion of the school setting in the implementation, attention to training, and quality of service delivery are indicators of users’ and caregivers’ satisfaction as well as device adoption [42-46].

### Contextualization Within Existing Literature

The development and initial evaluation of the vocal cord vibration switch have been described previously [13-15]; these previous evaluations were conducted over shorter time periods (0.5‐4.0 mo), primarily with adolescents, adults, and children older than 9 years and focused on the physical aspects of the Hummingbird, its reliability, and the ease of set-up for parents and teachers. The results from these earlier assessments informed a more formalized switch training process, the design of the neckband and its positioning, and the decision to use a questionnaire assessing family impact in the case series. In contrast, this case series measured the impact of long-term use in the child’s natural environment.

Fundamentally, our findings indicate that the Hummingbird can afford a pathway through which a child can access switch-controllable technology, inclusive of communication, mobility, and environmental devices. From the perspective of the International Classification of Functioning, Disability and Health for Children and Youth framework [3], our results suggest that via the Hummingbird (environmental facilitator), children’s vocal cords (body structures) can be accessed by amplifying vocal fold vibrations (body function) to expand independent, intentional switch control (activities) within their given setting (environmental participation conditions). Environmental conditions included barriers such as a child’s fatigue during sessions and school changes, as well as motivators such as music or a sibling joining in on a game. In turn, such technology-facilitated switch activations can augment children’s participation in communication, interpersonal interactions, education, and directing their own care (specific participation domains).

### Limitations

The assessment time points did not always follow the intended trajectory. For reasons of illness and family health issues, which reflect the reality of many families with children with multiple disabilities, some of the assessments were not conducted at the intended times and/or were not completed. Furthermore, some of the questionnaires were not completed at certain time points, resulting in missing data for the FIATS and the Criterion of Change for Zach. However, given the case series design, the results were interpreted individually rather than by making quantitative comparisons across participants. Furthermore, in instances where 1 assessment was missed, data from other time points were available, permitting nonetheless a temporal view of children’s and families’ experiences. Future studies may consider a different measure of family impact catered more toward children who are developing basic communication skills, such as expressing preferences.

For some data collection questionnaires, 2 different respondents (ie, mother and teacher) provided the assessment for a given child participant, based on who was present in the assessment session and where it took place (home or school). For both the response effort and QUEST 2.0 measures, scores were provided by a child’s mother at 1 session and a teacher at the subsequent session. This may have confounded the potential temporal changes in an outcome. Nonetheless, we arrived at a comprehensive assessment of the Hummingbird by deploying a variety of measures to capture the perspectives of multiple key informants in the training, use, and assessment phases of our protocol. Subsequent studies may consider mixed methods designs and associated integrated analyses that allow for informant-specific data sources [47].

Usage reporting was varied. Mothers’ reports regarding the usage of both the child’s prestudy device and the Hummingbird were unstructured (Table 3). As a result, the usage data varied in terms of how mothers reported use by units of time. To overcome this limitation, future research may consider invoking automatic logging of Hummingbird usage to objectively capture time and duration of use, the number of activations, and the functional application.

### Conclusions

Across home and school environments, the Hummingbird was appropriate for 3 school-aged children with complex communication needs as indicated by their low response effort, high scores on switch efficacy and immediacy of reinforcement, and strong caregiver satisfaction. The impact was generally favorable in familial and communication realms for the 2 participants with complete data. Participants encountered unanticipated health, family, and school challenges during the study, which necessitated modifications to the device and the implementation protocol. Future studies should consider a more expansive set of impact measures in light of these real-world circumstances.

## Abbreviations

AAC: augmentative and alternative communication
ATDP: Assistive Technology Delivery Protocol
FIATS: Family Impact of Assistive Technology Scale
ISO: International Organization for Standardization
PCERT: Pictorial Children’s Effort Rating Table
QUEST: Quebec User Evaluation of Satisfaction with Assistive Technology
